# Lymphoma of the Sublingual Gland: Clinical, Morphological, Histopathological, and Genetic Characterization

**DOI:** 10.3389/fsurg.2020.581105

**Published:** 2020-11-06

**Authors:** Lars Iversen, Patrick Rene Gerhard Eriksen, Simon Andreasen, Erik Clasen-Linde, Preben Homøe, Irene Wessel, Christian von Buchwald, Steffen Heegaard

**Affiliations:** ^1^Department of Ophthalmology, Rigshospitalet-Glostrup, Glostrup, Denmark; ^2^Department of Pathology, Rigshospitalet, Copenhagen, Denmark; ^3^Department of Otorhinolaryngology Head & Neck Surgery and Audiology, Rigshospitalet, Copenhagen, Denmark; ^4^Department of Otorhinolaryngology and Maxillofacial Surgery, Zealand University Hospital, Køge, Denmark

**Keywords:** lymphoma, sublingual glands, salivary glands, extranodal marginal zone lymphoma, mantle cell lymphoma, follicular lymphoma, head and neck

## Abstract

**Background:** Lymphoma of the sublingual gland is rare, representing 1% of all salivary gland lymphomas. In this case report, we present three new cases and compare them to previously published cases, with the aim of characterizing the clinical, morphological, histopathological, and genetic features of this type of malignancy.

**Materials and Methods:** We provide a clinical description of three cases along with a characterization of the microscopic features, including morphology, and immunohistochemistry. In addition, we analysed possible cytogenetic rearrangements with the use of fluorescence *in situ* hybridization (FISH).

**Results:** Case 1: A 61-year-old male presenting with a painless swelling of the floor of the mouth diagnosed as extranodal marginal zone lymphoma (EMZL) of the left sublingual gland. The patient is alive with no evidence of disease after his fourth treatment regimen following several relapses. Case 2: A 68-year-old female with a prior history of mantle cell lymphoma (MCL) presenting with a tender swelling of the left sublingual gland as well as the right submandibular gland. The lesions were diagnosed as relapsing MCL. The patient died of unrelated causes after 18 months of treatment. Case 3: A 75-year-old female presenting with a swelling of the floor of the mouth diagnosed as follicular lymphoma (FL) of the left sublingual gland. The patient received chemotherapy along with radiotherapy and was still alive 10 years after the diagnosis.

**Conclusion:** The three cases of sublingual gland lymphomas presented in this case report resemble lymphomas of other major salivary glands. The clinician should be aware of this type of malignancy and that the clinical presentation may not differ from benign lesions or other more common malignancies in this location.

## Introduction

Lymphomas are a group of malignancies, which arise from lymphocytes. Usually these are situated in lymphoid tissues but can also be found as an extralymphatic disease. Currently, the World Health Organization Classification recognizes more than 40 different types of lymphoma based on characteristic histology and genetic hallmarks each with a specific locational preference (1). Lymphomas are the third most common malignancy in the head and neck region after squamous cell carcinoma and adenocarcinoma (2), and the major salivary glands are the third most common site for extranodal lymphoma in the head and neck region followed by the ocular adnexa and sinonasal region (3). The Waldeyer's tonsillar ring is also a common site for head and neck lymphomas to arise but in recent literature, this site is not categorized as extranodal. Primary lymphomas of the salivary glands are rare, representing ~1.7–3.1% of all salivary gland neoplasms. These are distributed with 79% in the parotid glands, 18% in the submandibular glands, 2% in the minor salivary glands, and 1% in the sublingual glands (4). In the parotid gland, the most frequent types of lymphomas are extranodal marginal zone lymphoma (EMZL), follicular lymphoma (FL), and diffuse large B-cell lymphoma (DLBCL) (5), with EMZL being especially predominant in patients with Sjögren's syndrome (6). The detection of certain chromosomal translocations by fluorescence *in situ* hybridization (FISH) is used as a diagnostic tool and especially in EMZL genetic rearrangements vary in frequency according to the primary anatomical site of disease establishing a link between genetics and homing of malignant lymphocytes (7). Cytogenetically in salivary gland EMZL, the most common translocations detected are t(14;18)(q32;q21)-*IGH/MALT1* and t(11;18)(q21;21)-*API2/MALT1* (1).

The standard treatment of salivary gland lymphomas includes chemotherapy and/or radiotherapy (RT) depending on the lymphoma subtype. A surgical biopsy of salivary gland lymphomas is performed in order to establish a histological diagnosis. Lymphoma subtype, age of the patient, and stage of the disease, all influence the prognosis of parotid gland lymphomas, with increasing age and stage of disease resulting in a poorer prognosis (5). Additionally, transformation of indolent lymphomas to more aggressive subtypes, usually to DLBCL or high-grade B-cell lymphomas, further decreases the prognosis.

Due to the rarity of sublingual gland lymphomas, little is known about the distribution, presentation, histological subtypes, treatment, and prognosis of this patient group. Here, we present three new cases and characterize their clinical, morphological, histopathological, and genetic profile and compare them to previously published cases.

## Materials and Methods

The Danish national pathology data bank (PatoBank) was screened for all cases of lymphomas of the sublingual gland since 1980, resulting in four cases of alleged sublingual gland lymphoma. One case was excluded due to inability to fulfill the diagnostic criteria for lymphoma. Sublingual gland specimens and medical records from the remaining three patients were collected from the respective pathology, hematology and ENT-departments. If possible, staging was performed using the Cotswolds-modified Ann Arbor classification and the AJCC Lugano classification (8). Primary lymphoma of the sublingual gland is defined as no systemic involvement or involvement of other organs at the time of work-up and no prior history of lymphoma. Secondary lymphoma is defined as concurrent systemic disease or involvement of other organs at the time of work-up and/or prior history of lymphoma.

### Validation of Diagnoses

Formalin-fixed and paraffin-embedded (FFPE) tissue from the three sublingual gland lymphomas were cut and stained with haematoxylin and eosin (HE), periodic acid-Schiff (PAS), and Alcian blue. Immunohistochemistry was performed on a Ventana Benchmark Ultra platform (Ventana Medical Systems, Tucson, AZ, USA) as previously described (9). The following antibodies were applied: CD3, CD5, CD10, CD20, CD23, CD79α, cyclin D-1, BCL2, BCL6, MUM-1/IRF4, PAX5, SOX11, Ki 67, and κ and λ light chains. Positive and negative controls were included on each slide.

FISH was performed using the break-apart probes for *Bcl-2, Bcl-6, C-MYC*, and specific probes for *IGH/MALT1* and *IGH/CCND1* rearrangements, according to the manufacturers' protocol using the HYBrite platform (Abbott Molecular). After hybridization nuclei were counterstained with DAPI II (ZytoVision). One hundred nuclei were counted, and only nuclei where the entire nuclear membrane could be visualized were scored. Cut-off value was defined as 10%. Examination of the slides and validation of the diagnoses were performed by a specialized hematopathologist.

### Ethics

The study was approved by the local scientific ethics committee (Journal no. H-16023080) and the Danish Data Protection Agency (Journal no. P-2020-587). Written informed consent was obtained from the individuals for the publication of any potentially identifiable images or data included in this article.

## Results

### Case 1

**Figure 1:**
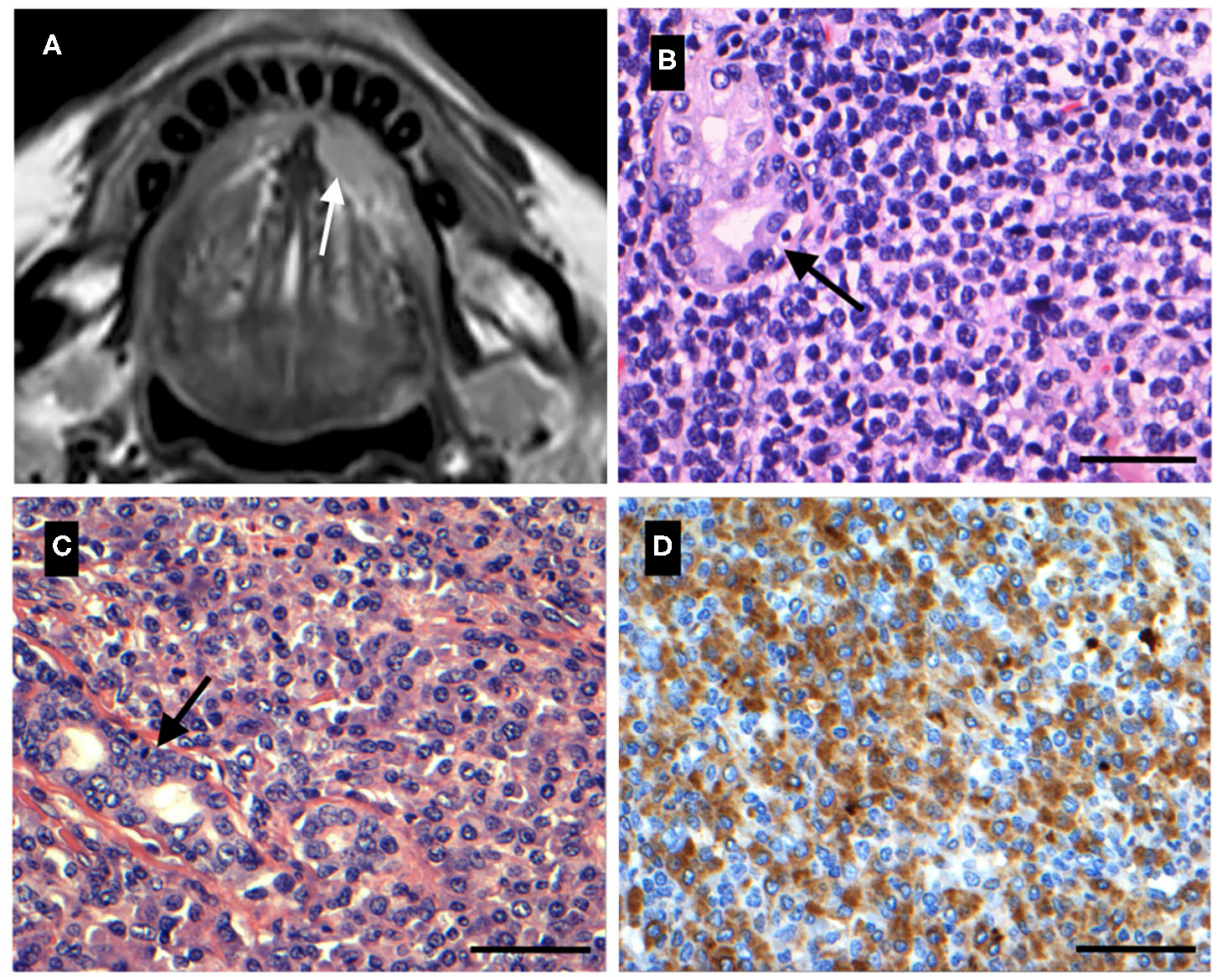
**(A)** A 68-year-old female with relapse of known Mantle cell lymphoma (MCL) resulting in swelling of the left sublingual gland. Magnetic resonance imaging (MRI) showing a tumor of the left sublingual gland (white arrow). **(B)** Histological image of the sublingual gland MCL **(A)**. Salivary gland tissue is infiltrated by lymphoid tumor tissue and small tumor cells are seen with multiple membrane bound nucleoli. Ductal structures are seen (black arrow) (HE). **(C)** A 61-year-old male with 1-month history of painless swelling of the floor of the mouth. Tumor tissue was composed of neoplastic lymphoid cells with irregular nuclei and plasmacytic differentiation, consistent with a diagnosis of extranodal marginal zone lymphoma (EMZL) of the left sublingual gland. Scattered ductal structures are seen (black arrow) (HE). **(D)** Immunohistochemistry of the sublingual gland EMZL **(C)** showing positive reaction for lambda light chain. Scale bar = 50 μm.

A 61-year-old male was seen in the ENT-department as an outpatient with a 1-month history of a painless swelling of the floor of the mouth. Previous medical history was unremarkable except for a Warthin's tumor of the parotid gland and prostate hypertrophy. Clinical examination revealed a two-centimeter mobile process in the left sublingual area (Table 1) and no enlarged lymph nodes in the head and neck region. The process was surgically excised in local anesthesia. Subsequent histological examination showed a well-defined tumor infiltrating salivary gland tissue with resulting destruction of the acinar architecture. Tumor tissue was composed of small homogeneous lymphoid cells with irregular nuclei and plasmacytic cell-differentiation among scattered ductal structures (Figure 1C). Immunohistochemically, case 1 showed positive reaction on the tumor cells' surface for Bcl-2 and CD20, and the proliferation marker Ki67 showed a proliferation index estimated to 20%. Furthermore, both lymphocytes and plasma cells showed lambda light chain restriction (Figure 1D). Finally, FISH was performed showing an IGH/MALT1 translocation (16% of the cells showing a signal), consistent with a diagnosis of EMZL of the sublingual gland. Subsequent bone marrow biopsy revealed lymphoma involvement and computed tomography (CT) did not show involvement of other sites. Accordingly, the lymphoma was Ann Arbor stage IVA and AJCC stage IV. The patient received chlorambucil for 1 year until complete remission. Three years later, the patient presented with a 2-month history of dry cough, night sweats, and an involuntary weight loss of 3–5 kg. CT scan revealed processes in the lungs, mediastinal-, and inguinal lymph nodes. Core needle biopsy from the right lung confirmed relapse of the EMZL. The patient received 8 series of rituximab, cyclophosphamide, vincristine, and prednisolone (R-CVP) until complete remission, with only one marginally enlarged lymph node remaining near the aorta, followed by a 2-year-period of maintenance therapy with rituximab. Three years later, the patient relapsed presenting with a subfascial tumor below the left infraorbital margin, resulting in three series of rituximab and bendamustine followed by RT (24 Gy in 12 fractions) until complete remission. Five years later, the patient again presented with night sweats and fatigue. Positron emission tomography (PET)-CT scan revealed multiple pathological lymph nodes above and below the diaphragm, and biopsies confirmed relapse of the EMZL with transformation to DLBCL. The patient received five series of rituximab, cyclophosphamide, hydroxydaunorubicin, oncovin, and prednisolone (R-CHOP) and subsequent PET-CT showed complete remission. The patient is currently scheduled for regular follow-up examination with diagnostic imaging and clinical visits.

**Table 1:** Demographic, clinical presentation, subtype, stage, treatment, and outcome of previously published sublingual gland lymphomas.

**Study**	**Age/sex/laterality**	**Clinical presentation**	**Subtype**	**Stage AA/AJCC**	**P/S**	**Treatment**	**Clinical course**
Takahashi et al. (12)	67/M/R	Swelling	EMZL	NA	P	Surgery + RT + chemotherapy (NS)	D, 3 yrs
Honda et al. (14)	71/F/B	Hard, elastic, mobile swelling	EMZL	NA	NA	RT	NA
Honda et al. (14)	63/F/R	Hard, elastic, mobile swelling	EMZL	NA	NA	Surgery	NA
Honda et al. (14)	82/F/L	Hard, elastic, mobile swelling	EMZL	NA	NA	Chemotherapy (NS)	D, 2 yrs
Yoshiba et al. (11)	64/M/L	Diffuse, firm, painless swelling	EMZL	I/IE	P	Surgery	NED, 36 mo
Makihara et al. (19)	81/M/R	Fibrous, painless swelling	EMZL	I/IE	P	Surgery	NED, NA
Present case 1	61/M/L	Painless, mobile swelling	EMZL	IVA/IV	S	Surgery + chlorambucil + RT	NED, 16 yrs
Rockacy et al. (16)	72/F/R	Painless swelling, normal overlying mucosa	MCL	IVA/IV	S	Surgery + observation	A, 1 yr
Hayashi et al. (15)	82/M/B	Elastic, hard, painless swelling	MCL	IV/IV	S	RT + rituximab	D, 3 yrs
Abukrian et al. (13)	70/M/B	Swelling	MCL	IV/IV	S	Rituximab-bendamustine	NED, 11 mo
Present case 2	70/F/L	Tender swelling, normal overlying mucosa	MCL	IVB/IV	S	Surgery + ibrutinib	DOC, 40 mo
Law and Leader (10)	58/F/R	Painless swelling	FL	IV/IV	S	Surgery + chlorambucil	NA
Williams et al. (18)	74/F/L	Firm, tender swelling, ulcerated mucosa	FL	IIIEA/IV	S	Surgery	NA
Present case 3	75/F/L	Mobile swelling, no redness, or wounds	FL	IA/IE	P	Surgery + CHOP + RT	NED, 10 yrs
Gleeson et al. (20)	NA	NA	SLL^*^	I/IE	P	Surgery	A, 34 mo
Schwartz-Arad et al. (17)	72/M/R	Asymptomatic swelling	LL^†^	NA	NA	Surgery + RT	A, 6 mo
León et al. (21)	44/M/NA	NA	DLBCL	NA	NA	NA	D, NA

*A, Alive; AA, Ann Arbor; AJCC, American Joint Committee on Cancer lugano staging system; AWD, Alive With Disease; B, Bilateral; CHOP, Cyclophosphamide, Hydroxydaunorubicin, Oncovin, and Prednisolone; D, Dead; DLBCL, Diffuse Large B-Cell Lymphoma; DOC, Dead of Other causes; EMZL, Extranodal Marginal Zone Lymphoma; F, Female; FL, Follicular Lymphoma; L, Left; LL, Lymphocytic Lymphoma; M, Male; MCL, Mantle Cell Lymphoma; mo, months; NA, Not Available; NED, No Evidence of Disease; NS, Not Specified; P, Primary; R, Right; RT, Radio Therapy; S, Secondary; SLL, Small Lymphocytic Lymphoma; yrs, years*.

* *leukemic infiltration*.

*not otherwise specified*.

### Case 2

A 68-year-old female was referred from an ENT specialist practitioner to the ENT-department due to multiple enlarged cervical lymph nodes located bilaterally and an involuntary weight loss of 10 kilograms over the last 6 months. There was no significant previous medical history. Fine needle aspiration biopsy (FNAB) suggested lymphoma and subsequent histological examination of an excised lymph node between level two and three from the left side of the neck confirmed the diagnosis of mantle cell lymphoma (MCL). PET-CT and bone marrow examination further demonstrated bone marrow involvement. Accordingly, the lymphoma was staged as Ann Arbor IVB and AJCC stage IV. The patient received eight series of R-CHOP followed by a scheduled 2-year-period of maintenance therapy with rituximab. Twelve months into maintenance therapy the patient developed a tender swelling of the right submandibular- and left sublingual gland. On clinical examination, a one-centimeter process with normal overlying mucosa was found in the left sublingual gland (Table 1) along with a similar process in the right submandibular gland. Ultrasound of the left sublingual- and right submandibular gland revealed two hyperechoic one-centimeter tumors. Further examination and ultrasound of the neck revealed no enlarged lymph nodes. A magnetic resonance imaging (MRI) of the head and neck was performed (Figure 1A). PET-CT revealed a process of the sigmoid colon, but the bone marrow examination showed no signs of infiltration. MCL relapse was confirmed by subsequent excision and histological examination of the sublingual- and submandibular gland, as well as histological examination of a biopsy specimen taken from the sigmoid colon via colonoscopy. Histological examination of the excised sublingual gland showed salivary gland tissue infiltrated by lymphoid tumor cells appearing as blastoid cells with multiple membrane bound nucleoli (Figure 1B). Mitotic figures were seen in several of these cells. Immunohistochemistry showed positive reaction in the tumor cells' nuclei for PAX5, cyclin D1, and SOX11 and on the tumor cells' surface for CD5 and the proliferation index was estimated to >80%. Furthermore, flow cytometry detected 70.1% clonal B-cells positive for CD5, CD10, CD19, CD20, CD43, and CD79 and with lambda light chain restriction. FISH demonstrated an IGH/CCND1 translocation (65% of the cells showing a signal), consistent with a diagnosis of MCL. Ibrutinib was initiated but the patient died 18 month later due to advancing heart failure and declining performance status.

### Case 3

A 75-year-old female was referred from an ENT specialist practitioner to the ENT-department due to a swelling of the floor of the mouth. Previous medical history was unremarkable. Clinical examination revealed a 1.5-centimeter mobile process at the left caruncle covered by intact mucosa (Table 1) and no enlarged lymph nodes in the head and neck region. FNAB of the lesion was inconclusive, thus the lesion was surgically excised in general anesthesia. Subsequent histological examination showed tumor tissue infiltrating closely packed salivary gland tissue. The tumor tissue was composed of lymphoid cells extending in a follicular growth pattern, consisting of a mixture of medium-sized irregularly shaped cells with indented nuclei and larger blastoid cells with multiple nucleoli. Approximately 15–20% of the tumor cells were centroblasts. Immunohistochemistry showed positive reaction on the tumor cells' surface for CD10, CD20, and Bcl-2 and in the tumor cells' nuclei for Bcl-6 and a proliferation index of 20%. Accordingly, case 3 was diagnosed as a grade II FL. Subsequent CT scan and bone marrow examination showed no signs of further dissemination of the disease and the lymphoma was classified as Ann Arbor stage IA and AJCC stage IE. Treatment included three series of CHOP followed by RT (26 Gy in 13 fractions). The patient went into full remission with no evidence of disease 10 years after the diagnosis, and the patient is still alive.

## Discussion

In this case report our research group presents three rare cases of sublingual gland lymphomas, significantly contributing to the number of cases reported worldwide. Table 1 provides an overview of demographic, clinical presentation, subtype, stage, treatment, and outcome of the present three cases along with previously published sublingual gland lymphomas (10–21). Overall, the most common clinical presentation was a painless swelling of the floor of the mouth with no signs of affected overlying mucosa (Table 1). Other salivary gland tumors may have a similar clinical presentation, and thus, there are no clear clinical distinguishable signs between benign lesions (e.g., pleomorphic adenomas) or malignant lesions (e.g., carcinomas, adenocarcinomas) of the sublingual gland and sublingual gland lymphomas. This has also been reported to be the case in parotid gland lymphomas (22). Sublingual gland lymphomas presenting as painful were more uncommon, with one MCL being reported as tender and one FL presenting as a tender swelling covered by ulcerated mucosa (Table 1). Differential diagnoses to painful swellings of the salivary glands include swelling caused by sialolithiasis or sialadenitis.

Including present and previous cases of sublingual gland lymphomas (Table 1), the most frequent histological subtype encountered was EMZL (41.2%), followed by MCL (23.5%), and FL (17.6%). This distribution is similar to the distribution in the parotid and lacrimal gland, with EMZL being the most frequent lymphoma subtype in these locations as well, accounting for 27.9 and 37%, respectively (5, 23). The primary genetic event in MCL is the t(11;14)(q13;q32) translocation between an *IGH* gene and the gene encoding cyclin D1 (*CCND1*). This gene rearrangement is seen in more than 95% of the cases, and thus serves as an important information in the diagnostic work-up of MCL. In contrast, multiple chromosomal translocations are associated with EMZL with frequencies of the translocations depending on the anatomical site. The t(14;18)(q32;21)/*IGH-MALT1* translocation is most commonly seen in salivary gland, ocular adnexa, and orbital EMZL (1), which shows that a genetic disposition in the lymphocytes lead to specific homing to and colonization of certain tissues (7). A feared complication to indolent lymphomas such as EMZL and FL is the risk of transformation to a more aggressive subtype, most commonly DLBCL or high-grade B-cell lymphoma characterized by a *MYC* and *Bcl-2* and/or *Bcl-6* translocation (so called double/triple hit lymphoma) and poor prognosis (24).

In general, RT is the treatment of choice for low stage indolent extranodal lymphomas, such as EMZL and FL, with the role of surgery largely being limited to diagnostic purposes. Systemic chemotherapy is indicated for more aggressive or advanced staged extranodal lymphomas, with RT primarily being used as adjuvant or palliative therapy (25). For asymptomatic patients with early stage FL, a watch and wait strategy is a widely accepted approach in regards to the initial management of the disease (26), though, ~2–3% per year of all follicular lymphomas transform to a more aggressive subtype (27). While single therapy RT is an accepted treatment for FL, newer studies suggest that the addition of rituximab to RT could further improve outcome in patients with early stage FL (28, 29). RT is also considered as a standard therapy for early stage EMZL, generally with a good prognosis (30). With more advanced staged EMZL, R-bendamustine has been proven to be both an effective and well-tolerated therapy regimen (31). In the case of advanced staged MCL, especially regarding elderly patients, who are not eligible for autologous stem-cell transplantation, chemotherapy is the treatment of choice. In these situations R-CHOP followed by rituximab maintenance therapy is a possible treatment regimen (32).

Stage and clinical course of previously published cases of sublingual gland lymphomas were not fully available, making it difficult to describe the prognosis of lymphomas located to this location (Table 1). Prior studies on lymphomas of other major salivary glands found that age, stage of disease, treatment, and lymphoma subtype influenced the prognosis, with EMZL being among the subtypes with the highest overall survival (OS) (5). In a recent study, 248 patients with EMZL of the parotid and submandibular glands were analyzed, and a median OS of 18.3 years and a progression-free survival (PFS) of 9.3 years were found (33). Marginal zone lymphomas of other extranodal sites have also been proven to have a favorable outcome, with reports of a 5-year OS of 75% for EMZL of the lacrimal gland (23). On the contrary, transformation of indolent lymphomas to more aggressive subtypes may result in an unfavorable prognosis. A PFS of 14 months and an OS of 42 months have been reported for indolent lymphomas undergoing secondary transformation after a preceding diagnosis (34).

In conclusion, we present three cases of sublingual gland lymphoma and characterize their clinical, morphological, histopathological, and genetic profile and compare them to previously published cases. The most common lymphoma subtype in this location is EMZL, but both MCL and FL has been reported as well. This is similar to the distribution found in the lacrimal gland and other major salivary glands, where EMZL is the dominating subtype as well. Clinical presentation of sublingual gland lymphomas may also resemble that of lymphomas located to other major salivary glands. In the assessment of a painless swelling of the sublingual gland, lymphoma should be considered as a differential diagnosis to gland inflammation, benign lesions, and other more common malignancies in this location such as carcinomas and adenocarcinomas, especially in patients with prior history of lymphoma or leukemia. The condition of the patient, along with the stage and subtype of the lymphoma, should be taken into consideration when choosing a treatment regimen. Outcome of the present three cases was not uniform but reflected the stage and subtype of the lymphoma. The limited number of cases included in this study makes it difficult to draw certain conclusions on the prognosis of sublingual gland lymphomas. In the future, further cases should be presented so to establish valid prognoses, progression rates, and treatment efficacy for lymphomas in this location.

## Data Availability Statement

All datasets generated for this study are included in the article/supplementary material.

## Ethics Statement

The study was approved by the local scientific ethics committee (Journal no. H-16023080) and the Danish Data Protection Agency (Journal no. P-2020-587). Written informed consent was obtained from the individuals for the publication of any potentially identifiable images or data included in this article.

## Author Contributions

EC-L: validation of histological diagnoses. SA and PE: analysis of FISH. LI: first draft. All authors conception and design, critical revision for intellectual content, and approval of the final version and accountable for all aspects of the work.

## Conflict of Interest

The authors declare that the research was conducted in the absence of any commercial or financial relationships that could be construed as a potential conflict of interest.
